# Sputum Smear and Culture-negative Tuberculosis with Associated Pleural Effusion: A Diagnostic Challenge

**DOI:** 10.7759/cureus.3513

**Published:** 2018-10-29

**Authors:** Muhammad U Asghar, Sanwal S Mehta, Hira A Cheema, Ravikaran Patti, William Pascal

**Affiliations:** 1 Internal Medicine, New York University Langone Medical Center, New York, USA; 2 Internal Medicine, Maimonides Medical Center, Brooklyn, USA; 3 Internal Medicine, Markham Stouffville Hospital, Markham, CAN; 4 Pulmonology, Maimonides Medical Center, Brooklyn, USA

**Keywords:** smear and culture negative tb, pleural effusion

## Abstract

Tuberculosis (TB) is an important cause of morbidity and mortality in the United States. Due to the unpredictable or nonspecific nature of its clinical presentations, TB can be a diagnostic challenge for physicians. In 2013, 23% of reported TB cases were culture-negative in the United States; in New York City, this was approximately 27%. The increasing number of sputum smear- and culture-negative TB patients is a serious concern because misdiagnosis and delayed treatment can lead to increased morbidity and mortality and increased infectious transmission. We report a case of a 26-year-old-female recent immigrant, who was initially managed for community-acquired pneumonia but was later found to have TB with complicated pleural effusion, despite having multiple smear- and culture-negative sputum specimens, Xpert Mycobacterium tuberculosis (MTB)/resistance to rifampin (RIF) assay (real-time polymerase chain reaction (PCR)) and pleural fluid analysis. She improved clinically on anti-tuberculosis therapy and, later, the diagnosis was confirmed by pleural biopsy.

## Introduction

Tuberculosis (TB) is a highly contagious infection caused by the aerobic, non-motile bacillus Mycobacterium tuberculosis (MTB) and is an important cause of morbidity and mortality worldwide. In New York City, there is a high prevalence of pulmonary TB, especially in the foreign-born population with approximately 27% of TB cases reported to be culture-negative (CX –ve) [1]. In 2013, the Center for Disease Control (CDC) has reported a 23% CX -ve rate for TB cases in the United States [2]. We report a case of a 26-year-old-female, a recent immigrant from Serbia, who was initially managed for community-acquired pneumonia but was later found to have tuberculosis with complicated pleural effusion, despite having multiple smear- and culture-negative sputum specimens.

## Case presentation

**Figure 1:**
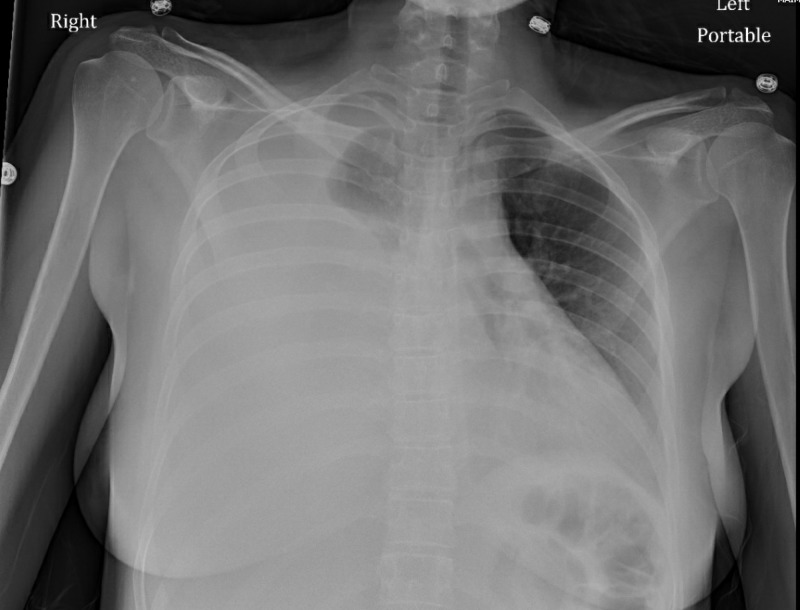
Significant right-sided pleural effusion

**Figure 2:**
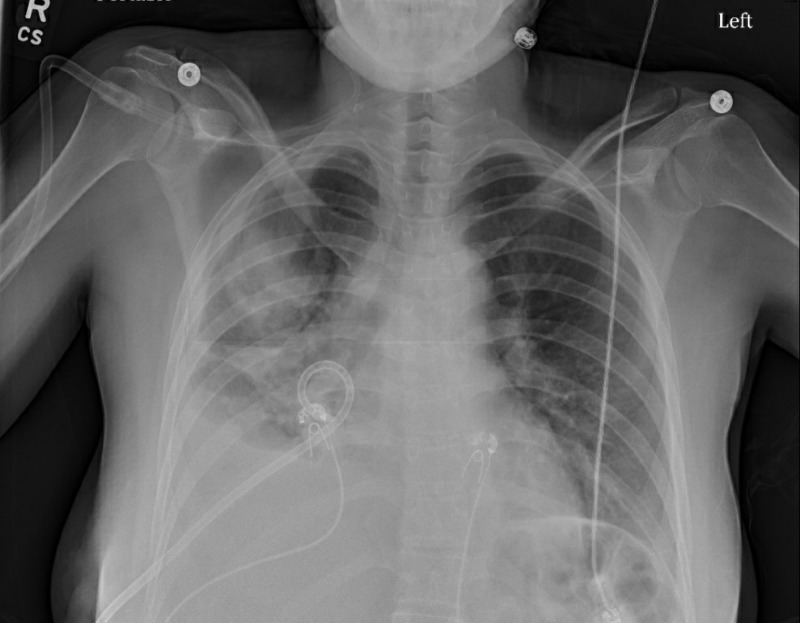
Right-sided chest tube placement and marked improvement of the effusion

 A 26-year-old-female, with no significant medical history and who had recently emigrated from Kosovo, Serbia, presented to the emergency department (ED) with complaints of a cough, chest pain, and shortness of breath for two weeks. She initially presented to urgent care, where she was diagnosed with pneumonia, prescribed azithromycin and sent home. Her symptoms did not resolve and she became more dyspneic despite oral antibiotics, which prompted her to come to the ED. Upon presentation, her vital signs were notable for a fever to 102 degrees Fahrenheit and a blood pressure of 92/60 mm Hg. The physical examination showed decreased breath sounds on the right on auscultation and dullness on percussion. The chest X-ray was notable for significant right-sided pleural effusion (Figure 1). Due to the size of the effusion and worsening respiratory status, emergent tube thoracostomy was performed, which drained the effusion significantly (Figure 2).

**Figure 3:**
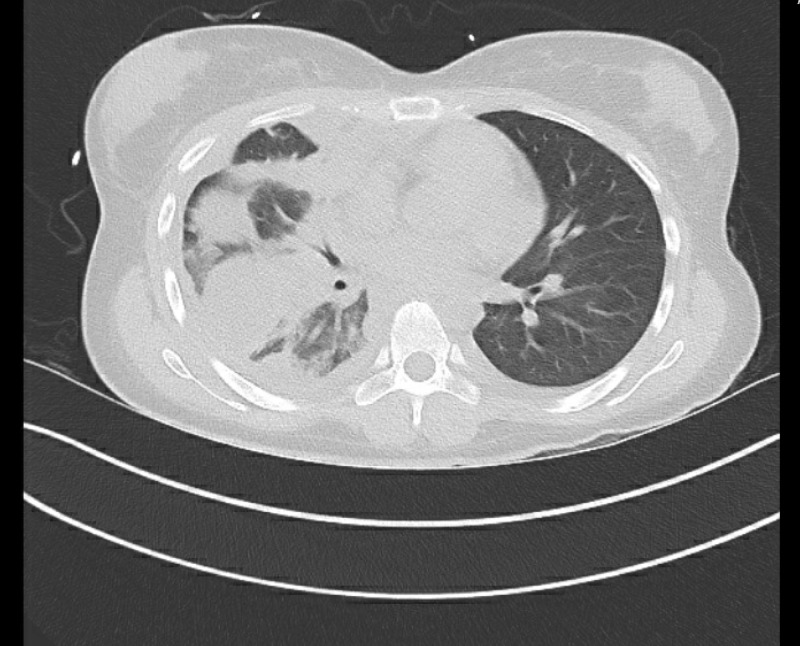
Computed tomographic scan done prior to video-assisted thoracoscopic surgery (VATS)

The patient was started on ceftriaxone and doxycycline for presumed pneumonia. Pleural fluid showed lactate dehydrogenase (LDH) 314 IU/L (compared with serum LDH 129 IU/L), and protein 5.2 g/L (compared with serum protein 5.5 g/L), indicative of exudative pleural effusion. Adenosine deaminase level was 1.7 units/L (N: 0-9.4 units/L) and a white blood cell (WBC) count of 1673 with 61% lymphocytes. Due to suspicion for TB, three acid-fast bacilli (AFB) sputum smear and cultures, collected eight hours apart, with one early morning sample, were sent. All the three sputum specimens came back negative for AFB. Even the first sputum cultures after six weeks were negative. Three samples were sent for an MTB/RIF assay (real-time PCR), which were also negative. Repeat chest X-ray showed decreased opacity in the right mid to upper chest with an appearance of increased effusion at the right lateral base. The computed tomographic (CT) scan of the chest was positive for pleural thickening and multiloculated pleural effusion (Figure 3).

Due to high suspicion for TB, the patient underwent right video-assisted thoracoscopic surgery (VATS) with decortication and biopsy. The histology from the pleural rind and a pleural nodule showed necrotizing caseating granuloma. At this point, the data were consistent with a diagnosis of pulmonary TB and the patient was placed on airborne isolation and started on standard anti-tubercular therapy with isoniazid, rifampin, pyrazinamide, ethambutol, and pyridoxine. Liver function tests were normal before initiating the therapy. After a week of therapy, the patient showed significant improvement in her respiratory symptoms. The Department of Health (DOH) was notified. As per their recommendations, three additional sets of sputum were sent for AFB, all of which came back negative, including a negative second set of sputum cultures six weeks later. The patient was discharged per DOH guidance, as she was not contagious. She continued anti-tubercular therapy as per standard protocol with close monitoring by DOH and outpatient infectious disease follow-up. The pleural biopsy from VATS was initially negative for gram stain and later came back positive for Mycobacterium tuberculosis, confirming the diagnosis of tuberculosis.

## Discussion

Mycobacterium tuberculosis (MTB) can be transmitted through the generation of airborne droplets during coughing, sneezing, or even speaking by a person with pulmonary or laryngeal tuberculosis. These droplets are inhaled and subsequently phagocytosed by macrophages in the alveoli. This results in a cascade of events, leading to either the successful resolution of infection followed by latent TB or progression to active pulmonary disease. Pulmonary tuberculosis is most often associated with the reactivation of latent infection but can also occur as a manifestation of primary infection. Pulmonary TB can form subpleural caseous foci, which subsequently rupture and complicate the disease process, leading to the development of pleural tuberculosis [3]. Pleural TB generally results from a late hypersensitive reaction caused by Mycobacterium tuberculosis antigen. Even a small number of bacilli in the pleural space can cause a significant inflammatory reaction. The mechanism is T-lymphocyte mediated, as these cells produce inflammatory cytokines that stimulate macrophages to form granulomas. This inflammatory process subsequently increases the vascular permeability and results in the formation of pleural exudates [4]. Moreover, radiographic imaging, such as computed tomography, has demonstrated that 40% of pleural and pulmonary TB cases are concomitant lesions rather than separate disease entities [5].

**Figure 4:**
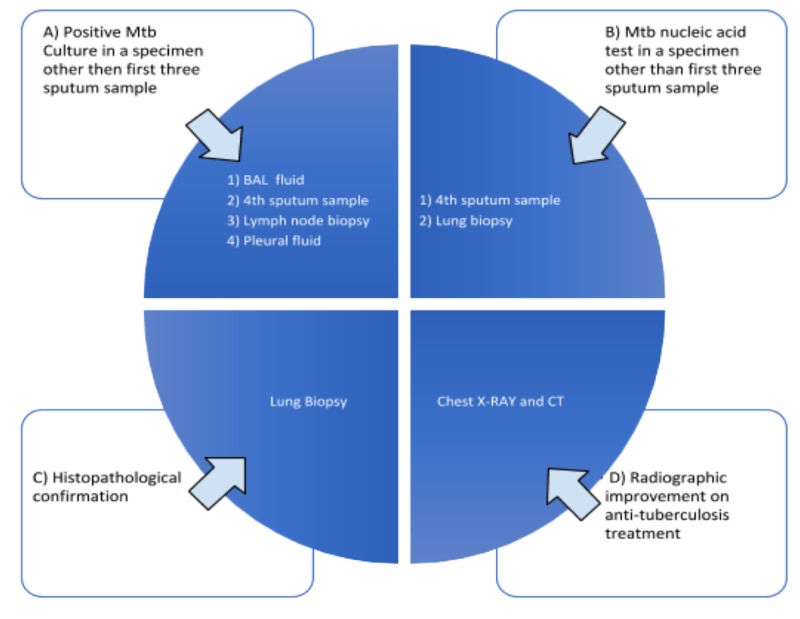
Confirmation methods for tuberculosis among sputum culture-negative patients

The diagnosis of pulmonary TB is classically made by sputum gram stain and culture along with the clinical and radiographic findings. Sputum smear is a rapid test to detect the presence of acid-fast bacilli (AFB) but a single sputum test lacks sensitivity. One of the reasons for low sensitivity is the fact that 10,000 microorganisms/ml are required for AFB to be seen on microscopy [6]. Furthermore, sputum culture requires 10 to 100 AFB/ml to confirm the diagnosis of TB [6]. The gold standard for the diagnosis of TB is culture or nucleic acid amplification assay. The Xpert MTB/RIF assay is a PCR test that can identify both Mycobacterium tuberculosis (MTB) and rifampicin resistance, with cultures typically being the more sensitive method for the diagnosis [7-8]. Culture-negative TB (CX -ve TB) is defined as no growth of MBT in the first three sputum samples, and it can be further investigated by the methods listed below in the diagram (Figure 4).

Studies have shown that combinations of diagnostic modalities can increase accuracy in diagnosing TB. Miro et al. found in a retrospective study that evaluated respiratory secretion samples collected by bronchoscopy for TB detection that adding the transbronchial biopsy increased diagnostic accuracy from 96% to 100% [9]. While sputum culture is the gold standard for diagnosis, many developing countries still rely on sputum smear as the diagnosis of active TB. Inadequate case detection is one major reason for the high burden of TB, especially in developing countries, as many cases are smear-negative and hence go undetected, further increasing the burden of TB.

Furthermore, smear- and culture-negative patients often present atypically, with less frequent cough, hemoptysis, and radiographic abnormalities compared to smear-positive or culture-positive patients [10]. Another study showed that CX- TB patients were more likely to have a chest CT as compared to CX+ patients and that lung cavitation is less frequently observed in culture-negative patients [11]. Importantly, a human immunodeficiency virus (HIV) infection dramatically changes the development of tuberculosis infection, as the risk of TB doubles within the first seven years of HIV infection due to a rapid decline in TB-specific T helper cells [12]. HIV-infected patients are at increased risk of developing reactivation TB and accelerate the progression of TB following a subsequent exposure. Considering the high burden and dissemination of TB infection, an HIV test was performed in our patient, which was negative.

## Conclusions

Physicians should not rely only on sputum smears and culture-negative results to exclude the diagnosis of TB but also consider advanced investigative modalities in patients with a high clinical likelihood of TB.
